# Synthesis and Antiproliferative Activity of Ammonium and Imidazolium Ionic Liquids against T98G Brain Cancer Cells

**DOI:** 10.3390/molecules171213727

**Published:** 2012-11-22

**Authors:** Nagendra Kumar Kaushik, Pankaj Attri, Neha Kaushik, Eun Ha Choi

**Affiliations:** Plasma Bioscience Research Center, Kwangwoon University, Seoul 139701, Korea; E-Mails: kaushik.nagendra@kw.ac.kr (N.K.K.); chem.pankaj@gmail.com (P.A.); neha.bioplasma@gmail.com (N.K.)

**Keywords:** ionic liquid, T98G brain cancer cells, HEK non-malignant cells

## Abstract

Four ammonium and imidazolium ionic liquids (ILs) have been synthesized and screened against the T98G cell line (brain cancer) and HEK normal cells. Treatment induced metabolic cell death (MTT), growth inhibition, clonogenic inhibition were studied as cellular response parameters. Treatment with ILs enhanced growth inhibition and cell death in a concentration dependent manner in both the T98G and HEK cell lines. At higher concentrations (>0.09 mg/mL) the cytotoxic effects of the ILs were highly significant. An inhibitory effect on clonogenic capacity was also observed after cell treatment. Amongst all ILs; IL **4** (BMIMCl) exhibited potent activity against T98G brain cancer cells. Despite potent *in-vitro* activity, all ILs exhibited less cytotoxicity against the normal human HEK cells at all effective concentrations.

## 1. Introduction 

In the race to synthesize new pharmceutical drugs, ionic liquids (ILs) have attracted a great deal of attention amongst in the scientific community due to their variety of potential pharmaceutical applications. ILs represents a big success for industrial and engineering chemistry at the beginning of the 21st century. ILs are defined as thermally stable salts synthesized by combining organic cations with a wide variety of anions. The huge interest in ILs has led to a broad and diverse range of ions known to support IL formation. One of the most appealing features of ILs for pharmaceutical applications is that they are highly customisable materials that can be specially made with pre-selected characteristics by varying the cations and anions of which they are comprised. Combinations of different cations and anions result in various ILs which provide a wide range of hydrophobicity/hydrophilicity, acidity/basicity, viscosities, *etc.* [1,2]. IL strategies can take advantage of the dual nature (discrete ions) to realize enhancements which may include controlled solubility (e.g., both hydrophilic and hydrophobic ILs are possible), bioavailability or bioactivity, stability, elimination of polymorphism, new delivery options (e.g., slow release or the IL-API as ‘solvent’), or even customized pharmaceutical cocktails [3]. ILs having an unique protective metabolic roles can act as an antioxidants [4], protect macromolecules, enhance protein folding and regulate cell volume [5,6]. ILs have also attracted the scientific community’s due to their pharmaceutical properties such as antimicrobial [7,8,9,10,11,12], antiseptic or antifouling actions [13], acetylcholinesterase (AChE) inhibition [14], AMP deaminase inhibition [15], delivery of anti-inflammatory drugs [16], local anesthetic [3], anti-nociceptive [3], anticholinergic [3] and anticancer activities [13,17], and in protein formulations [18,19,20,21]. Enzymes suspended in the ILs could be reused three times, with less than 10% loss of activity per cycle without influencing enantioselectivity [22]. Based on these interesting properties, ILs appear ready to provide a new research outlook in the field of medicinal chemistry. However the toxicity of some ILs has not been explored much in applications where this property can be desirable and lead to a variety of pharmaceutical applications [23]. Carson *et al.* have reported the broad spectrum antibioflim activity of 1-alkyl-3-methylimidazolium chloride ILs against a variety of clinically important microbes [24]. Therefore, ILs with their tunable properties and toxicities could potentially be designed as anti-cancer, anti-viral and other therapeutic agents [13]. If a therapeutic response were seen, then the major advantage of ionic liquids would be in tuning their toxicity while tailoring the physio-chemical and pharmacological properties necessary for the desired therapeutic application [13]. 

The present work is in continuation of our search for biological significant properties of ILs based on extensive studies reported earlier by Malhotra and Kumar [13,17]. In the present study, the anticancer activity of four ILs against T98G brain cancer cells was described. Cytotoxicity of the synthesized ILs on normal cells were assessed using the HEK cell line, which resembles developing neuron and neuronal stem cells and is also mentioned as a good model for neuroscience studies [25]. Based on this background, we have used HEK normal cells as a model for brain normal cells in this study. 

## 2. Results and Discussion 

### 2.1. Synthesis 

**Figure 1:**
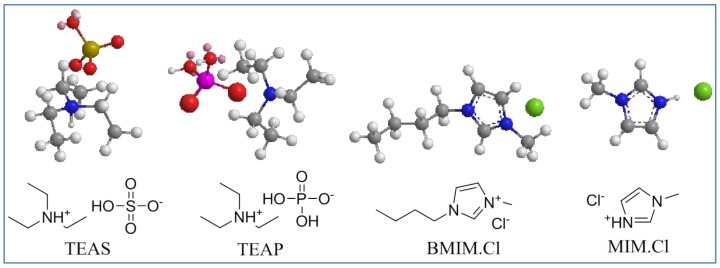
Structure of ammonium and imidazolium ILs. Brown, white, red, blue, pink, golden and green colored balls represent carbon, hydrogen, oxygen, nitrogen, phosphorus, sulphur and chlorine atoms, respectively. Small pink balls represent the lone pairs on oxygen atoms.

We have synthesized and studied the anticancer effects of ammonium and imidazolium ILs. The structure of ILs **1**, **2** and **3** are clearly supported by their ^1^H-NMR and microanalysis data. Figure 1 shows the prepared ILs, triethylammonium sulfate (TEAS, **1**), triethylammonium phosphate (TEAP, **2**), 1-methylimidazolium chloride ([MIM]Cl, **3**) and 1-butyl-3-methylimidazolium chloride ([BMIM]Cl, **4**). 

### 2.2. MTT Assay 

**Figure 2:**
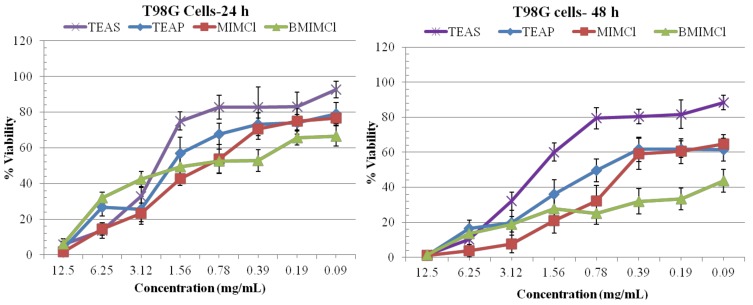

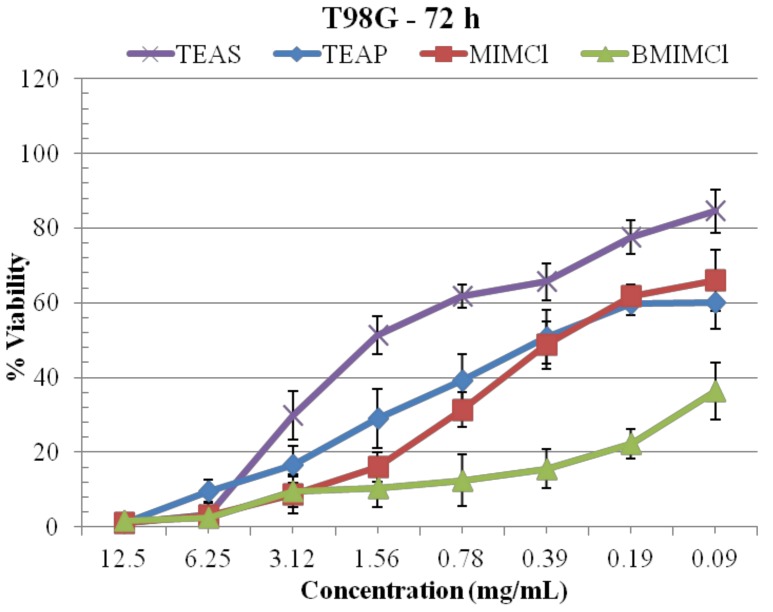
% Viability evaluated from MTT assay on T98G brain cancer cells treated with ILs for 24, 48, and 72 h. All values are considered significant, when *p* < 0.05.

**Figure 3:**
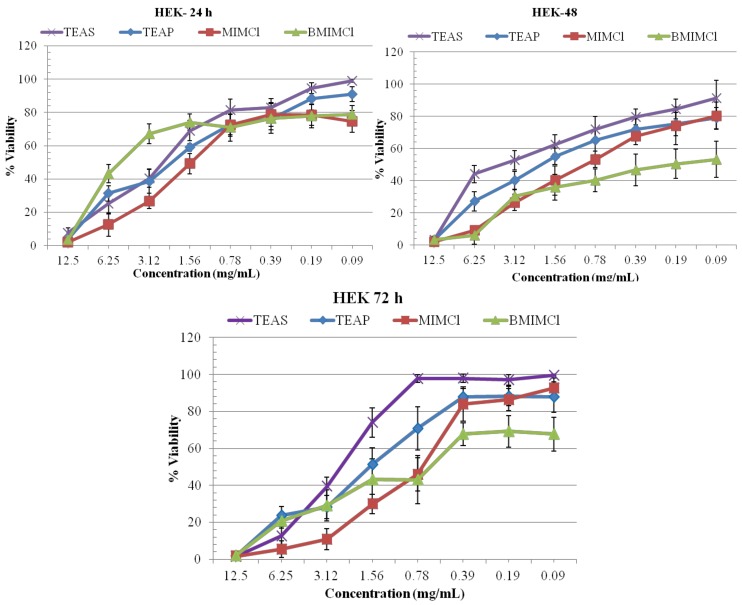
% Viability evaluated from MTT assay on normal HEK cells treated with ILs for 24, 48, and 72 h. All values are considered significant, when *p* < 0.05.

The 3-(4,5-dimethylthiazol-2-yl)-2,5-diphenyltetrazolium bromide (MTT) cell proliferation assay is widely accepted as a reliable way to measure the cell proliferation rates and cell death. The data obtained by the MTT assay show that ILs **1**, **2**, **3** and **4** had inhibitory effects on the growth of T98G and HEK cells in dosage-dependent manners (Figure 2 and Figure 3). ILs **1**, **2**, **3**, and **4** can inhibit 50% T98G cell growth (IC_50_) with a range of 0.78–0.09 mg/mL after 24, 48 and 72 h treatment (Figure 2). The maximum inhibitory effect was shown by IL **4** on T98G cells after 72 h of treatment, however IL **3** also effectively inhibits the growth of T98G cells at all the concentrations tested (0.78–0.09 mg/mL) and in time dependent manner (Figure 2). Overall IL **4** was the most potent one, having a significant inhibitory effect on T98G cells growth. All ILs are less toxic on HEK cells at all effective concentrations as compared with T98G cells (Figure 3). 

ILs **1** and **2** are less toxic to the HEK cells with 75–99% viability at range of 0.39–0.09 mg/mL concentration at all incubation time intervals, which further increased by decreasing concentration of ILs (Figure 3). For further growth kinetic studies, we have selected ILs **1** and **4** on the basis of its significant toxicity, against T98G brain cancer cells. 

### 2.3. Growth Kinetics Assay 

**Figure 4:**
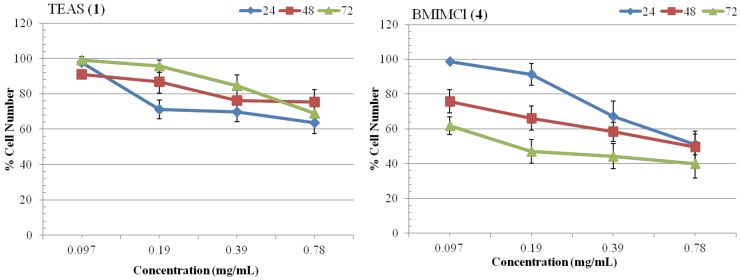
Growth kinetics of T98G cells at 24, 48 and 72 h after treatment by ILs at 0.097–0.78 mg/mL concentrations. Untreated cells are taken as control and all values given as mean (±SE) of three independent experiments, n = 3. All values are considered significant, when *p* < 0.05.

**Figure 5:**
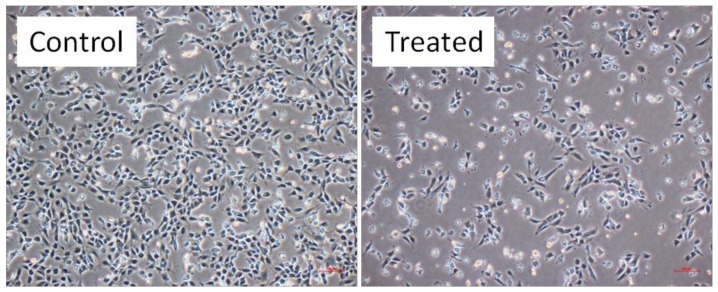
Morphology of T98G cells. treated with IL **4** at 0.19 mg/mL concentration after incubation for 24 h. Control is untreated cells.

Figure 4 shows the growth kinetics of T98G cells treated with ILs **1** and **4**. Cell proliferation kinetics have been studied at 24, 48, 72 h after IL treatment, following trypsinization and counting total cells per plate by using a trypan blue dye and a hemocytometer. Data obtained from the growth kinetics assay shows that the ILs **1** and **4** have an inhibitory effect on the growth of T98G cells. IL **1** shows this inhibitory effect only in a concentration dependent manner, however IL **4** shows an inhibitory effect in a concentration as well as in an incubation time dependent manner (Figure 4). It was also noted that the cells exposed to 0.097 mg/mL concentration of IL **4** shows less significant (*p* > 0.04) growth inhibitory effects than those treated with 0.19, 0.39 and 0.78 mg/mL concentration (*p* < 0.04). Maximum effect was shown by 0.78 mg/mL concentration of IL **4**; where it inhibits the growth of cells up to 61% at 72 h after treatment and its viability range was 39.9% (*p* = 0.03). In the case of 0.19 and 0.39 mg/mL exposures of IL **4**, we found 56–53% cells death (*p* < 0.04) at 72 h after treatment. Whereas in case of IL **1**, we found only 16–37% cell death (*p* < 0.05) at 0.39 and 0.78 mg/mL concentrations, at all incubation time after treatment. Overall, growth kinetics assay results also shows that IL **4** is having maximum inhibitory effect on T98G cells. Cell morphology analysis have revealed that size and density of viable cells were also affected by treatment of IL **4** (Figure 5). 

### 2.4. Clonogenic Assay 

**Figure 6:**
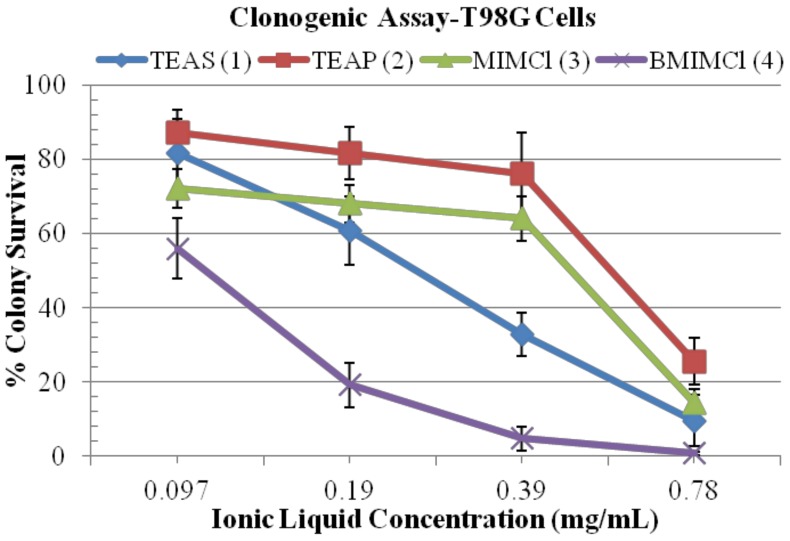
Effect of ILs on the colony-forming capacity or clonogenic survival of exponentially growing T98G cell lines studied by a clonogenic assay. Data presented are mean values from three independent observations, n = 3. All values are considered significant, when *p* < 0.05.

The clonogenic assay shows the effect of ILs **1**, **2**, **3** and **4** on the colony-forming capacity and survival of exponentially growing T98G cells. We have used the clonogenic assay to confirm our growth inhibition results with the ILs. The clonogenic assay or colony formation assay is an *in vitro* assay based on the ability of a single cell to grow into a colony. Only a fraction of seeded cells retain the capacity to produce colonies after cytotoxic drug treatment. It observed in Figure 6, the surviving fraction of T98G cells has been drastically decreased after treatment with ILs. 

All IL treatments enhanced cell death and also inhibited the colony-formation capability in the T98G cell population in a concentration dependent manner. After treatments with different concentrations (0.097–0.78 mg/mL), the surviving fractions of T98g cells declined, as evidenced by the reduction in the number of colonies formed. Even at low doses (0.097, 0.19 and 0.39 mg/mL) exposure to IL **4** results in a significant decline (*p* < 0.04) in colony survival and their corresponding % survival were found to be 56, 19.2 and 4.8, respectively. However, a more significant drastic decline (*p* = 0.04) in their survival fraction could be observed after exposure to 0.78 mg/mL treatment of IL **4** and their survival fraction were found to be 0.8. IL **1** also shows higher inhibitory effect on T98G cells, and at doses of 0.097, 0.19, 0.39 and 0.78 mg/mL exposure showed % survival rates of 81, 60.8, 32 (*p* = 0.046) and 9.6 (*p* = 0.007), respectively. This shows that these treatments are significantly inhibiting the colony formation capabilities of brain cancer cells at all the dosages. The most drastic clonogenic inhibitory effect was found after treatment with 0.39 and 0.78 (*p* < 0.05) mg/mL concentrations of ILs. 

### 2.5. In Silico Pharmacokinetics 

For a molecule to be a potential drug, besides having a good biological activity, it must have good pharmacokinetic properties in biological systems. To access the pharmacokinetic profile of the synthesized molecules, we used well validated *in silico* tools: Osiris, Chemaxon and Catalyst. These tools have been validated with almost 7,000 drug molecules available on the market. 

**Figure 7:**
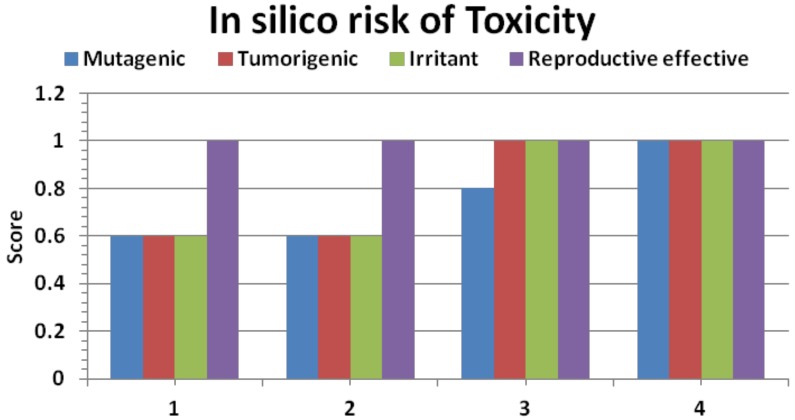
*In silico* drug safety analysis for ILs by the Osiris software. High score is an indicator of safe drugs (Score 1 is a highly safe drug indicator).

The analysis of theoretical toxicity risks for the ILs using the OSIRIS program shows that all ILs were less toxic and can be used as therapeutic molecules (Figure 7). 

As these ILs are considered for oral delivery, they were submitted to the analysis of Lipinski ‘rule of five’, druglikness and drug score by using the Catalyst software (Table 1). Our results pointed that all effective ILs fulfill this rule and their druglikness property such as molecular weight (118–199), LogP (0.12–1.5), nHBA (1–2), nHBD (0) and number of rotatable bonds (rotb) (0–3) were better than those of commercial drugs (Table 1). Finally, we evaluated all ILs as potential drugs by calculating druglikeness and drug-score. Druglikeness, which is related to the similarity with trade drugs (−0.35–−1.29). Drug score of all ILs were in the range of 0.128–0.560. Among the all ILs, **4** showed the best values of drug-score (0.56) with a lower toxicity risk, which points it out for further exploration. 

## 3. Experimental 

### 3.1. General 

The MTT (3-[4,5-dimethylthiazol-2-yl]-2,5-diphenyltetrazolium bromide), bisbenzimidazole derivative Hoechst-33342 (bisbenzimide(2-[4-ethoxyphenyl]-5-[4-methyl-1-piperazinylpiperazinyl]-2,5-bi-1*H*-benzimidazole)trihydrochloride), *N*-[2-hydroxyethyl]piperazine-*N*-[2-ethanesulfonic acid] (HEPES) buffer, propidium iodide (PI), ribonuclease-A (RNase-A), Tris-hydrochloride, propidium iodide (PI) were obtained from the Sigma Chemical Co. (Yongin, Korea). Trypsin-EDTA were obtained from the Gibco (Grand Island, NY, United States). Antibiotic-antimycotic solution and phosphate buffer saline (PBS) were obtained from Welgene (Daegu, Korea). Dulbecco’s modified phosphate buffered saline (PBS), Dulbecco’s modified eagle’s medium (DMEM), fetal bovine serum (FBS) were obtained from Hyclone (Logan, UT, USA). Triethylamine, sulfuric acid, phosphoric acid, 1-methylimidazole and 1-chlorobutane was obtained from Sigma–Aldrich (Yongin, Korea). *1-methyl imidazolium chloride ([MIM]Cl*, **3**) were also obtained from Sigma–Aldrich (Yongin, Korea), are of high purity, were used without further purification. All solvents and acids used in the present study were of analytical grade and obtained from Sigma-Aldrich (Yongin, Korea). Instruments used in the present study are Synergy HT plate reader (Biotek, USA), Nikon Eclipse T*i* (Nikon, Japan), Jeol-500 NMR (Japan). 

### 3.2. Preparation of ILs ***1**–**4***

All the ammonium and imidazolium families of ILs were synthesized in our laboratory. The synthesis of ILs was described in our previous papers [26,27,28]. 

*Synthesis of triethylammonium sulfate (TEAS*, **1**). The synthesis of TEAS was carried out in a 250 mL round-bottomed flask, which was immersed in a water-bath and fitted with a reflux condenser. Sulphuric acid (1 mol) was added dropwise to triethylamine (1 mol) at 60 °C for 1 h. The reaction mixture was heated at 80 °C under vigorous stirring for 2 h to ensure that the reaction had proceeded to completion. The reaction mixture was then dried at 80 °C until the weight of the residue remained constant. The sample was analysed by Karl Fisher titration which revealed very low levels of water (below 70 ppm). The purity of the IL was estimated by ^1^H-NMR spectroscopy. The yield of TEAS was 198 g. ^1^H-NMR (CDCl_3_): 1.3 (t, 9H), 3.16 (m, 6H), 5.04 (s, 1H). Melting point: 90 °C. 

*Synthesis of triethylammonium phosphate (TEAP*, **2**). The synthesis of TEAP was carried out in a 250 mL round-bottomed flask, which was immersed in a water-bath and fitted with a reflux condenser. Phosphoric acid (1 mol) was added dropwise to triethyl amine (1 mol) at 70 °C for 1 h. The reaction mixture was heated at 80 °C under vigorous stirring for 2 h to ensure that the reaction proceeded to completion. The reaction mixture was then dried at 80 °C until the weight of the residue remained constant. The sample was analysed by Karl Fisher titration which revealed very low levels of water (below 70 ppm). The purity of IL was estimated by ^1^H-NMR spectroscopy. The yield of TEAP was 198 g. ^1^H-NMR (DMSO-*d*_6_): *δ* (ppm) 1.18 (t, 9H), 3.06 (m, 6H), 6.37 (s, 1H). Melting point: 92 °C. 

*1-Methyl imidazolium chloride ([MIM]Cl*, **3**). Please see 3.1 (General Section). 

*Synthesis of 1-Butyl-3-methylimidazolium Chloride ([BMIM]Cl*, **4**). To a vigorously stirred solution of 1-methylimidazole (1.25 mol) in toluene (125 mL) at 0 °C was added 1-chlorobutane (1.38 mol). The solution was heated to reflux at 110 °C for 24 h, after which it was placed in a freezer at −20 °C for 12 h. The toluene was decanted and the remaining viscous oil/semi-solid was recrystallized from acetonitrile and then repeatedly recrystallized from ethyl acetate to yield a white crystalline solid, which was dried *in vacuo* to give [BMIM]Cl in approximately 86% yield. ^1^H-NMR (400 MHz, DMSO-*d*_6_) δ: 10.54 (1H, s), 7.55 (1H, m), 7.40 (1H, m), 4.26 (2H, t, *J* = 7.3 Hz, 4.11 (3H, s), 1.82 (2H, m), 1.30 (2H, m), 0.89 (3H, t, *J* = 7.3 Hz). Melting point: 67 °C. 

### 3.3. Human Cell Culture 

T98G malignant (brain cancer) and Human Embryonic Kidney (HEK) non-malignant cells used in the present studies were purchased from the Korean Cell Line Bank (KCLB), Seoul, Korea. We cultured these cell lines in 75 cm^2^ culture flasks (SPL, Pocheon, Korea) using Dulbecco’s modified Eagle’s medium (DMEM) supplemented with 10% fetal bovine serum, 1% nonessential amino acids, 1% glutamine, penicillin (100 IU/mL) and streptomycin (100 µg/mL) according to the manufacturer’s instructions. All cultures were maintained at 37 °C, 95% relative humidity and 5% CO_2_. Prior to each cytotoxicity test, the cells were harvested using trypsin–ethylenediamine tetraacetic acid (EDTA)–PBS solution (with 0.25% trypsin–0.05 mM according to the distributor’s instructions) and diluted at a density of 10^5^ cells/mL for assays. Stock cultures were passaged every third day after harvesting the cells with 0.05% trypsin and seeding 8 × 10^3^ cells/cm^2^ in tissue culture flasks to maintain the cells in the exponential phase. All experiments were carried out in exponentially growing cells. The cell suspension was seeded into 24-well plates (SPL) at 100 µL/well, and incubated for approximately 20–24 h before tests in order to reach confluency. 

### 3.4. In Vitro Metabolic Viability Assay 

Cells were seeded in 24-well plates at a concentration of 10^4^ cells/well in 200 μL of complete media and incubated for 24 h at 37 °C in 5% CO_2_ atmosphere to allow for cell adhesion. All assays were performed in two independent sets of quadruplicate tests. Control group containing without treatment was run in each assay. Following after 24, 48 and 72 h of exposure of cells to IL, each well will be carefully rinsed with 200 μL PBS buffer. Cytotoxicity were assessed using the MTT (3-[4,5-dimethylthiazol-2yl]-2,5-diphenyltetrazolium bromide) assay. MTT solutions 20 μL (5 mg mL^−1^ dd H_2_O) along with 200 μL of fresh, complete media were added to each well and plates were incubated for 3 h [29,30,31,32]. Following incubation, the medium were removed and the purple formazan precipitate in each well were sterilized in 200 μL DMSO. Absorbances were measured using microplate reader at 570 nm and results were expressed as % viability which is directly proportional to metabolic active cell number. Percentage (%) viability were calculated as:

% Viability = OD in sample well/OD in control well × 100

### 3.5. Growth Kinetics Assay 

Cells were seeded at 8,000 cells/cm^2^ in 100 mm Petri disc or flask, and their proliferation kinetics studied at 24, 48, 72 h after treatment with the ILs, following trypsinization and counting total cells per flask/disc using a hemocytometer [29]. 

### 3.6. Clonogenic Assay 

Clonogenic assay or colony formation assay is an *in vitro* cell survival assay based on the ability of a single cell to grow into a colony. The colony is defined to consist of at least 50 cells. The assay essentially tests every cell in the population for its ability to undergo “unlimited” division [33]. The clonogenic assay is the method of choice to determine cell reproductive death after treatment with ionizing radiation, but we can also be used to determine the effectiveness of drug molecules. Only a fraction of seeded cells retains the capacity to produce colonies before or after treatment, cells will be seeded out in appropriate dilutions to form colonies in 1–3 weeks. After harvesting with 0.05% trypsin, 150–400 (depending on the treatment) cells will be plated 10–14 h before treatment in DMEM at 37 °C. Cultured cells will be treated with doses 20 to 100 ug/mL of ILs. After the treatment, cells will be incubated in dark under humidified, 5% CO_2_ atmosphere at 37 °C for 8–10 days to allow colony formation. Colonies will be fixed with methanol and will be stained with 1% crystal violet. Colonies of more than 50 cells will be counted and the surviving fraction (SF) will calculated. Clonogenic survival curves will be constructed from three independent experiments by least-squares regression fitting averaged survival levels. 

### 3.7. In Silico Pharmacokinetic Screening 

To evaluate pharmacokinetic profile descriptors such as cLog P (octanol/water partition coefficient) and Log S (water solubility) were calculated using the Osiris Property Explorer on-line system available at [34]. The ILs were submitted to *in silico* ADMET (absorption, distribution, metabolism, excretion, and toxicity) screening, using the Osiris program. Values of druglikeness are based on the occurrence frequency of each fragment of the molecule in commercial drugs while the drug-score evaluates the IL’s potential to qualify for a drug and is related to topological descriptors, fingerprints of molecular druglikeness, structural keys and other properties such as cLog P and molecular mass [35,36]. *In silico* theoretical safety analysis is also evaluated using the Osiris software, where a score of 1 means a drug is safe and a score <1 means a drug molecule is theoretically toxic for use. The pharmacokinetic profile, important for a good oral bioavailability of a IL, was also evaluated according to Lipinski’s ‘rule-of-five’ using the Catalyst and Chemaxon softwares, which analyse features that a drug should present to allow the absorption and permeation across the membranes and states molecular weight <500 Daltons (Da), calculated octanol/water partition coefficient (cLog P) < 5, number of hydrogen-bond acceptors (nHba) < 10, and number of hydrogen-bond donors(nHbd) < 5 as well as a fifth rule added later, which infers the number of rotatable bonds < 10 [37]. 

### 3.8. Statistical Analysis 

Data have been expressed as means ± SD. Statistical analysis was done by student T-tests. Results were considered significant when their *p*-value < 0.05. 

## 4. Conclusions 

The obtained results with ILs against human T98G brain cancer cells were compared with HEK non-malignant cells. These ILs were more efficacious on T98G cancer cells and less toxic to HEK non-malignant cells at effective concentrations. These ILs may be regarded as lead molecules for a new class of cytotoxic agents effective against T98G brain cancer cells with good drug delivery capability because of their small size and capable of chemosensitization. Finally we can conclude that ILs could have the broad dosage ranges of activity against the human T98G brain cancer cells. IL **4** shows significant toxicity on T98G cells and the least toxicity on normal Human Embryonic Kidney (HEK) cells at effective concentrations. IL **4** also inhibits the clonogenic capacity of T98G brain cancer cells in a concentration and time dependent manner. IL **4** presents the overall best parameters including: (a) high activity against T98G brain cancer cell line; (b) low cytotoxicity on HEK cells at effective concentrations; (c) low toxicity risks in *in silico* analysis; (d) good oral bioavailability according to the Lipinski ‘rule of five’; and (e) better druglikeness and drug-score values, nearly similar to commercial drugs. Studies on the mechanism by which the IL **4** induces antiproliferative effects on T98G brain cancer cell lines are ongoing. 

## Figures and Tables

**Table 1:** Pharmacokinetic parameters (Catalyst, Chemaxon and Osiris softwares).

ILs	nHba	nHbd	nrotb	MW	cLog P	Druglikeness	Drug Score
1	1	0	3	199	1.5	−1.29	0.130
2	1	0	3	199	1.5	−1.28	0.128
3	2	0	0	118	0.12	−0.35	0.560
4	2	0	3	174	1.14	−0.79	0.641
